# Novel Insights into the Structure and Reduction of Graphene Oxide: A Case of Thiourea

**DOI:** 10.3390/ma18225135

**Published:** 2025-11-12

**Authors:** Oksana Oskolkova, Viktoriya Gnatovskaya, Darya Trush, Elena Vylivok, Ekaterina Khomutova, Leonid Fershtat, Alexander Larin

**Affiliations:** 1 L. M. Litvinenko Institute of Physical Organic and Coal Chemistry, Donetsk 283048, Russia; gares@list.ru (O.O.); vicktoria.gnatovskaya@yandex.ru (V.G.); kurilenckodash@yandex.ru (D.T.); alena.viliwok@yandex.ru (E.V.); ek.khomutova75@yandex.ru (E.K.); 2 N. D. Zelinsky Institute of Organic Chemistry, Russian Academy of Sciences, Moscow 119991, Russia; fershtat@ioc.ac.ru

**Keywords:** graphene oxide, reduction, aerogel, thiourea, Raman spectroscopy

## Abstract

In this work, samples of reduced graphene oxide (rGO) were prepared by treating graphite oxide (GrO) with thiourea (TU) and ascorbic acid (AA). Aerogels rGO-TU and rGO-AA were prepared using the freeze-drying method and were analyzed using X-ray diffraction, FTIR and Raman spectroscopy, ^1^H and ^13^C NMR, TEM, and SEM-EDS. Based on the NMR, FTIR, SEM-EDS, and TEM data, GO with TU is reduced with simultaneous functionalization of its oxygen-containing groups. According to ^1^H and ^13^C NMR data, the reduction of GO occurred simultaneously with an interaction of the amino groups of thiourea with carbonyl groups on the graphene sheets, forming an imine bond. This is evidenced by the appearance of additional signals in the ^13^C spectrum of GO-TU samples in the region of 140–230 ppm. The Boehm titration method showed that the number of oxygen-containing groups in rGO-TU aerogels decreased by about five times compared to GO. However, thiourea interacts with the GO surface, most likely due to electrostatic interaction and hydrogen bonds. The adsorption capacity of rGO-TU aerogel with respect to methylene blue (MB) after 1440 min was 60.2 mg/g, while for rGO-AA it was 71.4 mg/g. This fact indicates the importance of optimizing GO reduction to increase the number of active sites.

## 1. Introduction

Three-dimensional (3D) carbon materials in the form of aerogels, hydrogels, foams, and sponges possess unique physicochemical and electrochemical characteristics. In particular, oxidized and reduced carbon-based materials have applications in water desalination technologies [1,2,3], drug delivery systems [4,5], gas sensors [6,7], oil–water separation [8], solar cells [9,10,11], and energy storage devices [12,13,14,15], among others. Although various approaches for graphite oxidation have been proposed, the methods for producing GO require additional improvements. Graphite is not only of significant scientific value as the primary form of oxidized carbon, but it also serves as an important technological platform for creating various derivatives and composites decorated with metal nanoparticles or heteroatoms covalently bonded to carbon (for example, sulfur, nitrogen, phosphorus, etc.). Recently, sulfur-modified carbon materials were found to be promising in the field of electrochemistry [16,17,18], for heterogeneous catalysis [19], as adsorbents for the extraction of ecotoxicants from aqueous media [20], and as sorbents for carbon dioxide [21].

One of the new techniques for improving the surface integrity of titanium alloys using electrical discharge machining (EDM) is the use of a powder mixed dielectric made of graphene and water [22]. Recently, the effect of the rGO content in the dielectric on the ionization of the plasma channel and the scattering of electrical discharges was analyzed [23]. The effect of powder mixed EDM on surface roughness and its integrity properties depends on the discharge current, pulse duration, electrode polarity, and the concentration (%) of rGO in the dielectric. The experimental results show that upon performing EDM with rGO, scattering of discharges occurs on the rGO flakes. This leads to a multiplication of discharges during a single pulse. In the study [24], the machinability of a new Nimonic alloy was studied using EDM by evaluating the material removal rate, tool wear rate, and surface roughness. The treatment was carried out using a conventional dielectric and a mixed dielectric containing GO nanoparticles (5 g/L). The microstructural study of the machined surface and sustainability, along with a detailed comparative analysis of the responses, assures the superiority of machining in a nano-GO mixed dielectric–Cu electrode environment.

Polymer composites with graphene and functionalized graphene have attracted particular attention in the last decade. Graphene, obtained by reducing graphene oxide, is most often used to create composites. Numerous studies have shown that even a small addition of a graphene component to a polymer matrix can significantly improve the mechanical and barrier properties of the material, as well as enhance its electrical and thermal conductivity and resistance to external influences [25].

Low-toxicity thiourea, which has three functional groups and is widely used in organic synthesis, is an attractive reagent for the reduction and functionalization of GO [26]. One such method for graphene production involves the reduction of graphene oxide using thiourea. The authors suggest that this approach is more environmentally benign compared to traditional toxic reducing agents, such as dimethylhydrazine, hydroquinone, and sodium borohydride, thereby offering a safer and more sustainable alternative [27]. Reduced graphene oxide (rGO) obtained via thiourea reduction (rGO-TU) exhibited conductivity of 635 S·m^−1^ and demonstrated excellent dispersion stability in both protonic and aprotic solvents. Moreover, rGO-TU possesses a relatively high selective adsorption capacity for gold, reaching a value of 833 mg·g^−1^ [28]. GO covalently functionalized with thiourea on a porous ceramic substrate demonstrates high hydrogen permeability while effectively rejecting methanol and small ions. This selective transport behavior makes the material a promising candidate for applications in gas separation, solvent purification, and seawater desalination [29]. It was shown in [30] that graphene oxide functionalized with thiourea and urea inhibits corrosion of a carbon steel alloy in an acid medium. The aim of this study is to synthesize rGO-TU and evaluate its adsorption capacity toward methylene blue.

## 2. Materials and Methods

### 2.1. Materials

The purified natural flake graphite used was the Formula BT 2935APH (Superior Graphite Co., Chicago, IL, USA, carbon content: 99.95%; particle size distribution: 90% of particles < 32 μm). All reagents were of analytical grade and used without purification. Purified water with a resistance of 18.25 MΩ·cm was used in all experiments. The chemical purity of thiourea and ascorbic acid was determined by Fourier transform infrared spectroscopy (FTIR). The FTIR spectra of thiourea and ascorbic acid corresponded to the spectra posted in the Spectral Database for Organic Compounds (SDBS).

### 2.2. Synthesis of Graphene Oxide (GO)

The limiting stage in graphite oxidation is the process of oxidant diffusion into the interlayer space of graphite; therefore, the classical method of exhaust gas synthesis using Hummer’s method has been modified, where 1 g of graphite was mixed with 0.5 g of NaNO_3_ and 23 cm^3^ of concentrated H_2_SO_4_. The resulting mixture was stirred for 20 min, then 3 g of KMnO_4_ was added; the mixture was kept at a temperature of 0 ± 2 °C for 1 h. Then, the reaction mixture was heated to 35–40 °C for 30 min. Next, 46 cm^3^ of distilled water was added to the mixture, heated to 90–95 °C, and kept at this temperature for 15 min. Then, 140 cm^3^ of chilled distilled water and 50 cm^3^ of a 3% aqueous H_2_O_2_ solution were added. The peroxide solution was prepared before starting the experiment by diluting it with water, having previously determined the density and exact concentration of the initial solution. The resulting graphite oxide (GrO) was separated from the reaction mixture by centrifugation and decantation. In the same way, the product was washed twice with 2.5% HCl solution, and then with distilled water until the neutral reaction of the rinsing waters. The obtained GrO was dried in a drying oven at 50 °C. Aqueous GO suspensions were prepared by dissolving a sample of dried GrO in distilled water. The resulting suspension was treated with ultrasound in a Codyson CD-4800 ultrasound bath (Shenzhen Codyson Electrical Co., Ltd., Shenzhen, China) (42 kHz, 70 W) for 30 min.

### 2.3. Reduction of Graphene Oxide

#### 2.3.1. Reduction by Thiourea (rGO-TU)

GO was reduced using thiourea as a reducing agent. A weighed portion of GO was placed in a thermostatic reactor, and distilled water was added (GO concentration 4 mg/cm^3^). The mixture was then ultrasonicated in a Codyson CD-4800 ultrasonic bath (42 kHz, 70 W) for 30 min to obtain a GO suspension. Next, a weighed portion of TU was added to the reactor (the mass ratio of GO:TM = 1:3). The interaction of GO with TU was carried out at 80 °C for 1 h. Then, the reactor was again placed in an ultrasonic bath for 1 h. The rGO-TU was a dense black hydrogel in the shape of the reactor.

#### 2.3.2. Reduction by Ascorbic Acid (rGO-AA)

Bulk GO was placed in a thermostatically controlled reactor, and distilled water was added (concentration of GO 7.8 mg/cm^3^). The mixture was then treated with ultrasound in a Codyson CD-4800 ultrasonic bath (42 KHz, 70 W) for 1 h to obtain a GO suspension. An ascorbic acid (AA) was added to the reactor (ratio GO:AK = 1:2). The interaction of GO with AA was carried out at 80 °C for 3–4 h. The resulting reduction product was a dense black hydrogel that follows the shape of the reactor.

### 2.4. Preparation of Aerogels Using Sublimation Drying

rGO aerogel was obtained by drying the synthesized material and triple-washing with distilled water hydrogels using the sublimation method in a lyophilic dryer EV-DF10A Top-press with manifold for 26 h (t = −65 °C, *p* = 0.1 Pa).

### 2.5. Material Characterization

#### 2.5.1. X-Ray Diffraction Phase Analysis (XRD)

The structural features of graphite, GO, and rGO were studied by XRD using a DRON-3 diffractometer (Burevestnik factory, St. Petersburg, Russia) with CuKα radiation, wavelength λα = 1.54181 Ǻ (U = 30 kV, I = 20 mA). The diffraction patterns were recorded in the angle range of 2Θ = 8.0–90° with a scanning rate of 1°/min. The positions of the peaks in the diffraction patterns were determined with an accuracy of 2Θ = 0.04°. The calculated and experimental values of the sliding angles 2Θ were estimated with an accuracy of (Δ 2Θ ± 0.05)°. The calculated and experimental values of the interplanar distances were estimated with an accuracy of (Δd(00l) ± 0.001) nm. To further describe the microstructure and find the profile parameters, the reflexes of various phases were approximated using the Voigt function. The Wolfe–Bragg Equation (1) was applied to reflection (002) to estimate the distance between the layers (d). The Scherrer Equation (2) with a constant K equal to 0.9 was used to estimate the average size of nanoparticles (D). Taking into account that the Scherrer equation with a constant K = 0.9 is applicable to spherical particles, it is possible to equate the value of the average size of nanoparticles, D, to the value of the average height of the layers and calculate, using Equation (3), the average number of graphene layers in a package, n.

2dsinΘ = Nλ,(1)

where d is the interplanar distance, nm; Θ is the sliding angle, °; N is the order of reflection; and λ is the wavelength, nm.

H = Kλ/βcosθ,(2)

where H is the average size of coherent scattering regions (domains, crystallites); K is the dimensionless particle shape coefficient (Scherrer constant); and β is the width of the Laue half-height reflex (in units of 2θ).

n = D/d,(3)

#### 2.5.2. Raman Spectroscopy

Raman spectra were recorded using a LabRam 300 system (Horiba Scientific Inc., Irvine, CA, USA) equipped with a microscope (50× and 100× magnification) and a TV camera. The excitation wavelength of the HeNe laser was 632.8 nm. The spectral resolution was 2 cm^−1^. The laser power used was in the range of 0.05–5 MW, depending on the sample type. The frequency bands were calibrated using the Si line at 520 cm^−1^ and the N_2_ gas lines at 2230 cm^−1^. Micromapping was performed with a spatial resolution of ~1 μm. The average crystal grain size in the sp^2^ region of GO was calculated using the following equation [31]:

(4) $$L_{a}\left(nm\right)=\;\frac{\left[2.4\times 10^{-10}\times {{(\lambda }_{i})}^{4}\right]}{\left[\frac{I_{D}}{I_{G}}\right]}$$

where La is the average grain size; I_D_ and I_G_ are the intensities of peaks D and G, respectively, in the Raman spectra; and λ_i_ is the laser wavelength (632.8 nm).

#### 2.5.3. Transmission Electron Microscopy (TEM)

The morphology and internal structure of carbon nanomaterials were studied using a JEM-200A microscope from JEOL (Tokyo, Japan). Samples for microscopy were formed on specially carbon-coated copper grids by placing a drop of the dispersion of the sample under study on the grid or by applying it using an ultrasonic sprayer. The image in the form of negatives was recorded on AGFA photographic film (CAMERA CE, orthochromatic line film, ISO 9001 [32] approved, Germany). After development and drying, the negatives were digitized using a SONY DSC-H5 camera (Sony Group Corporation, Tokyo, Japan) or a CanoScan 8800F scanner (Canon, Tokyo, Japan). An electron diffraction pattern for determining the crystalline structure of the sample was obtained by changing the focal area of the magnetic lens.

#### 2.5.4. Scanning Electron Microscopy (SEM) with Energy Dispersive X-Ray Spectroscopy (SEM-EDS)

The surface morphology and elemental composition of the nanomaterials were studied using a JSM-6490LV scanning electron microscope (JEOL Ltd., Tokyo, Japan) equipped with energy-dispersive X-ray spectroscopy equipment (INCA Penta FETx3 attachment, OXFORD Instruments, High Wycombe, UK). Microscope operating mode: accelerating voltage (U)–10, 20 kV, current (I)–(0.3…3.0) × 10^−10^ A, magnification—×50…×20,000, and contrast in secondary and reflected (backscattered) electrons. The samples were applied to a special substrate made of double-sided carbon tape. The remains of the samples that did not stick to the substrate were removed using an air stream. Then, the samples were placed in the microscope column for research. In the case of non-conductive samples, a conductive layer of aluminum was applied to the powder surface using the spraying method in a VUP-5 vacuum unit (Sigma-Aldrich, St. Louis, MO, USA). The elemental composition of the selected micro-areas of the studied object, the percentage ratio of the identified elements, and their distribution topography on the object during microscopic studies were determined using an INCA Energy-350 energy-dispersive X-ray spectrometer (Oxford Instruments, Abingdon, UK). The elemental composition of the selected microparticles of the object under study, the percentage of identified elements, and their topography of distribution on the object during microscope studies were determined using an energy dispersive X-ray spectrometer INCA Energy-350.

#### 2.5.5. Fourier Transform Infrared Spectroscopy (FTIR)

FTIR spectra of the samples were obtained on a Bruker Alpha spectrometer equipped with a deuterated triglycine sulfate detector in a KBr matrix containing about 2% of the material by weight. Measurements were made in a range from 400 to 4000 cm^−1^ with a wavenumber resolution of 4 cm^−1^, and the data are presented as the average of 16 scans for each sample.

#### 2.5.6. Adsorption Experiment

The methylene blue (0.007 g) was dissolved in 200 cm^3^ of hot distilled water. The resulting solution was cooled and placed in a 1000 cm^3^ measuring flask and brought to the mark with distilled water. The molar concentration of the working methylene blue (MB) solution was 0.02 mmol/dm^3^. The calibration graph was obtained using MB solutions with concentrations of 0.0025, 0.005, 0.01, and 0.015 mmol/dm^3^. The concentrations of the MB solutions were measured using a UV–vis spectrophotometer (Helios Gamma, Thermo Electron Corporation, Waltham, MA, USA) at a wavelength of 664 nm. The optical density measurement was carried out at a constant temperature and the same pH value. A constant pH value was ensured by using a potassium phthalate acid buffer solution with a pH value of 4.0. Buffer solution was prepared from buffer solutions and working pH standards (standard titer).

A certain mass of rGO aerogels (2 mg) was added to 10 mL MB (0.02 mmol/dm^3^) in a 50 mL conical flask, closed, and shaken for 5 min. After shaking, the suspension was transferred to centrifuge tubes and centrifuged for 5 min at 3000 rpm. The supernatant was carefully collected with a Pasteur pipette and transferred to a spectrophotometric cuvette for measurement of optical density.

The adsorption activity of the aerogel for the indicator was calculated using the equation:

(5) $$X=\;\frac{(C_{1}-C_{2})\times V}{m}$$

where C_1_ (mg/dm^3^) and C_2_ (mg/dm^3^) are the initial and residual concentrations of MB in the suspension, respectively. V(dm^3^) and m (g) are the volume of the MB solution and mass of the adsorbent, respectively.

#### 2.5.7. Boehm Titration

Three precise samples of GO weighing 0.1 g or rGO aerogels of 0.2 g each were placed in three round-bottomed flasks with a volume of 100 cm^3^, and 25.0 cm^3^ of water was added. The flasks were sealed with stoppers with exhaust pipes. The solutions were vacuumized and degassed for 5 min in an ultrasonic bath. Then, the flasks were opened and 25 cm^3^ of 0.1 M solution was added: sodium carbonate in the first, sodium bicarbonate in the second, and sodium hydroxide in the third. The flasks were then closed, vacuumized, and treated with ultrasound for 5 min. Hermetically sealed flasks were kept for 3 days. Next, the solutions were filtered through a paper filter, and 5 cm^3^ of filtrate was taken into flasks for titration, in which 50 cm^3^ of water and 5 cm^3^ of 0.1 M hydrochloric acid were previously placed. The determination of oxygen-containing groups was carried out using the method of potentiometric acid–base titration, and titrated with 0.1 M sodium hydroxide solution using a microburette with constant stirring on a magnetic stirrer. The titration was carried out in an inert gas atmosphere. The number of functional groups (n, mol) in GO or rGO was determined using Equation (6):

(6) $$n=\;C_{in}V_{in}-V_{in}\frac{C_{1}V_{1}-C_{2}V_{2}}{V_{3}}$$

where C_in_ is the initial concentration of the base, mol/dm^3^; V_in_ is the volume of the base solution in the flask, dm^3^; V_1_ and C_1_ are the volume and concentration of 0.1 M hydrochloric acid; V_2_ and C_2_ are the volume and concentration of the titrant, 0.1 M sodium hydroxide; and V_3_ is the aliquot, dm^3^.

The amount of oxygen-containing functional groups per 1 g of GO or rGO (X) was determined using the equation (mmol/g):

(7) $$X=\;\frac{n}{m}$$

where m (g) is the mass of the sample of GO or rGO.

#### 2.5.8. Solid-State NMR

Solid-state NMR (SSNMR) experiments were recorded on a Bruker AVANCE III WB 400 MHz spectrometer equipped with a 4.0 mm DVT MAS BB/HF probe (15 kHz) and a 2.5 mm DVT MAS BB/HF probe (35 kHz) (^1^H—400.1 MHz, ^13^C—100.6 MHz, ^81^Br—100.25 MHz). Samples were spun at 9–13.5 kHz (4.0 mm probe) at the magic angle (MAS) using ZrO_2_ rotors. SSNMR spectra from the samples were recorded at the natural abundance of the ^13^C isotope (1%), which usually requires long-term accumulation due to the weak signal. Due to the high electrical conductivity of the samples, to enable rotation of the MAS and avoid interference, the samples were diluted with neutral carrier (KCl was used) approximately 4–6 times, thoroughly ground, and mixed. ^1^H MAS spectra were recorded using a single-pulse sequence with a 30° pulse at 13.5 kHz (4.0 mm probe) and a recycle delay of 5 s. ^13^C solid spin-echo experiments were performed using a Hahn-Echo MAS synchronized pulse sequence using a 4.0 mm probe with a spinning rate of 10 kHz and a recycle delay of 10 s, short acquisition time of ~3.5 ms under high-power proton decoupling (HPDEC) conditions, typical spin-echo times of 95.5 us (1 rotor period at 10 kHz), high-power 90-degree ^13^C pulses of 3.0 us and 145 W with an RF field of 83.3 kHz, exponential apodization with LB ~ 300 for processing, and a wide spectral sweep (typically more than 0.6 MHz). ^13^C CP MAS spectra were recorded with a recycle delay of 5 s, acquisition time of ~5 ms, contact times of 3–4 ms at 10 kHz, and a wide spectral sweep (typically 0.6 MHz). The ^13^C under high-power proton decoupling (HPDEC) conditions was recorded using “spinal64”. Chemical shifts for ^1^H and ^13^C were relative to an external adamantane sample. The magic angle was calibrated precisely for the spinning side bands in the ^81^Br spectra of the KBr sample.

## 3. Results and Discussion

### 3.1. Morphology and Structure Characterization

**Figure 1:**
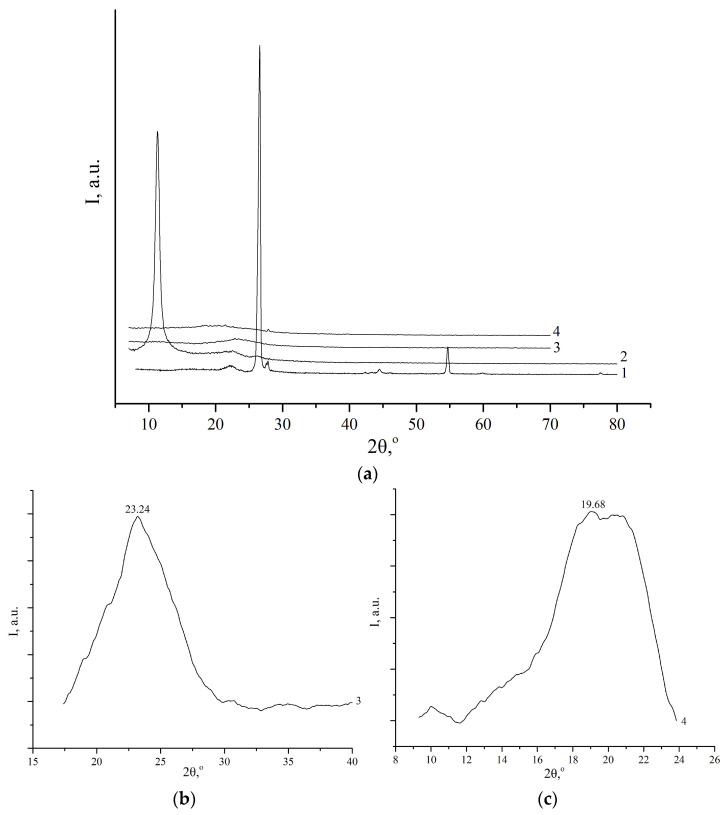
X-ray diffraction of initial graphite (1, (**a**)), graphite oxide (2, (**a**)), graphene oxide reduced by thiourea (3, (**a**,**b**)), and graphene oxide reduced by ascorbic acid (4, (**a**,**c**)).

The X-ray diffraction patterns of the initial graphite, GO, rGO-TU, and rGO-AA are shown in Figure 1.

For pure graphite, a peak of basal reflection (002) is observed at 2θ = 26.6°, which corresponds to an interplane distance of 0.34 nm (Table 1). After oxidation of the initial graphite, the reflection peak 002 shifted toward a smaller angle of 2θ = 11.41°, which corresponds to the layered structure of oxidized graphite with a distance between the layers of about 0.8 nm, and with the number of layers in the package of about 16–17. The low-intensity reflexes 2θ = 22.78° and 2θ = 26.36° belong to the phases of non-oxidized graphite and graphite that has lost its layered structure. According to the X-ray diffraction data of rGO-TU and rGO-AA, treatment of GO with thiourea or ascorbic acid leads to its recovery. However, the carbon ring frame does not fully restore its original graphite appearance and instead undergoes further distortion. This is evidenced by a pronounced broadening of reflection (002) in the range of 2θ = 18–25° with a d-distance of 0.38 nm and 0.45 nm (Table 1), which indicates a slight difference from samples of natural graphite (0.34 nm). The data are consistent with the work in [33]. It should be noted that for rGO-TU, the peak shifts toward large angles by 1.2 times compared to rGO-AA. This may indicate a more effective reduction.

**Figure 2:**
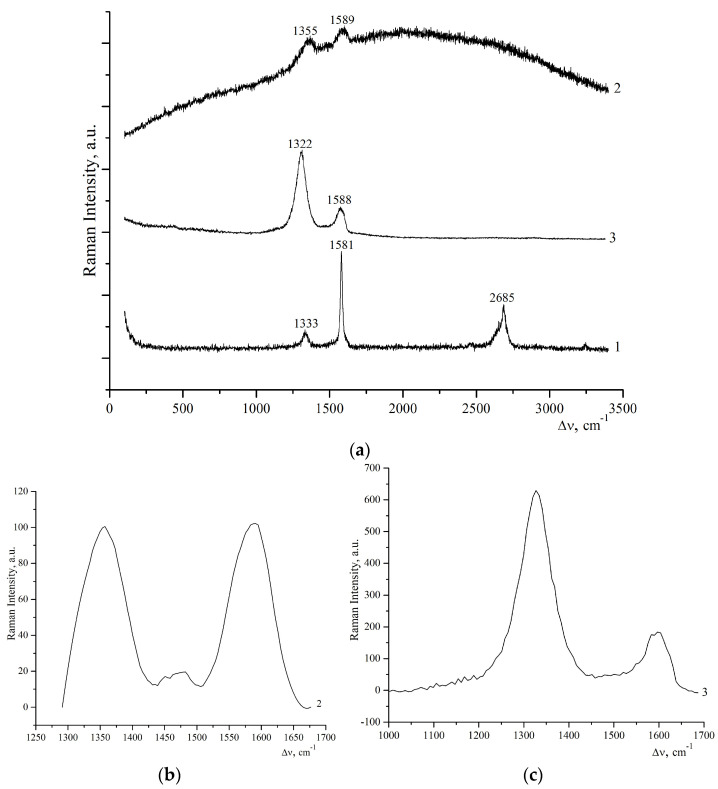
Raman spectra of initial graphite (1, (**a**)), graphite oxide (2, (**a**,**b**)), and graphene oxide reduced by thiourea (3, (**a**,**c**)).

The Raman spectra in Figure 2 for the prepared GO sample showed typical D and G peaks near 1355 and 1587–1600 cm^−1^, respectively [31,34]. According to the Raman spectroscopy data in Table 2, the increasing I_D_/I_G_ intensity ratio indicates that the introduction of oxygen-containing functional groups has disrupted the original graphite structure, and the size of the graphite crystal grains with sp^2^ domains has significantly decreased compared to the original graphite. The increase in the I_D_/I_G_ ratio may correspond to grafting of the thiourea amino group and the reduction of GO during thiourea modification [35]. These results are consistent with the findings of [36], where graphite oxide was simultaneously reduced and modified with ethylenediamine.

Thus, the crystallite sizes in oxidized graphite were approximately 39 nm, while in the original graphite they were approximately 265 nm. Upon reduction of GO with thiourea, the crystallite size of rGO-TU decreased compared to that of GO and was approximately 12–13 nm, while the I_D_/I_G_ intensity ratio increased, indicating an increase in the proportion of amorphous structures on one side and the removal of oxygen-containing functional groups at the edges of the rGO samples.

**Figure 3:**
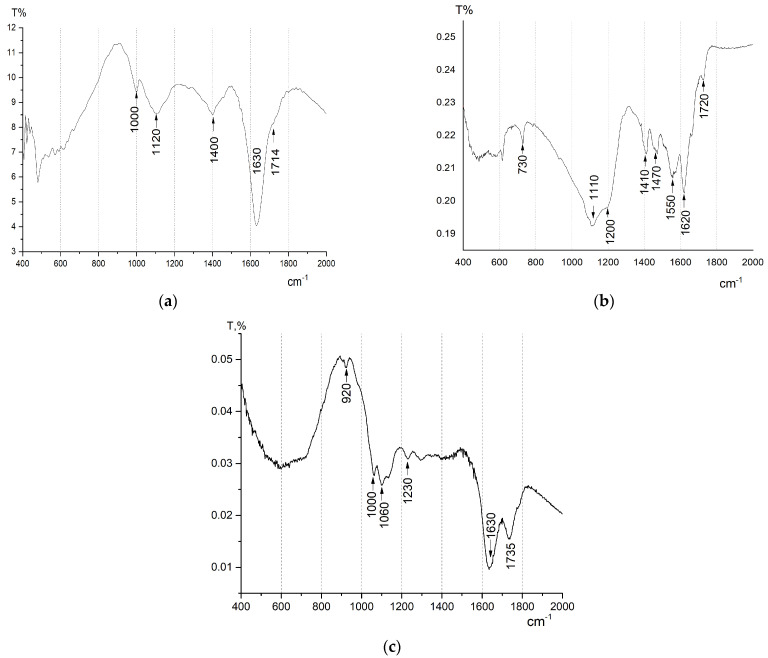
FTIR-spectra for GO (**a**), rGO−TU (**b**), and rGO−AA (**c**).

FTIR analysis (Figure 3a) showed that the synthesized GO samples contained no significant amounts of epoxy groups, as there are no absorption bands in the 870–930 cm^−1^ and 1240–1260 cm^−1^ regions [37].

The spectrum shows absorptions at 1000 cm^−1^, 1120 cm^−1^, and 1630 cm^−1^, which correspond to C–O stretching vibrations in alkoxy and hydroxy groups bound to carbon. A shoulder at 1714 cm^−1^ and absorption at 1400 cm^−1^ correspond to C=O stretching vibrations of carbonyl groups.

From Table 3, it can be seen that the number of oxygen-containing groups in rGO-TU decreased, which once again confirms the reduction of GO by thiourea.

**Scheme 1:**
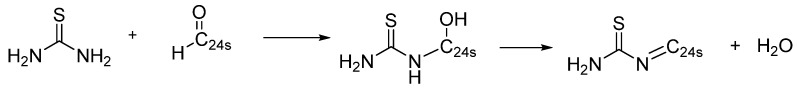
The plausible mechanism of interaction of the carbonyl group with thiourea.

However, the process of GO reduction with thiourea, according to [38,39], can proceed to simultaneous functionalization by using a reducing agent. Indeed, after reduction/functionalization of GO with thiourea, a new peak appeared in the FTIR spectrum of rGO-TU (Figure 3b) at ~1550 cm^−1^, corresponding to the C=N stretching vibration, which may indicate the formation of imine groups or conjugated C=N fragments as a result of the interaction of the amine groups of thiourea with electrophilic centers on GO. It should be noted that the rGO-AA FTIR spectra do not show absorption at 1550 cm^−1^, which further supports the assumption of a new bond formation in the rGO-TU. The absorption bands at 1470 cm^−1^ and 1410 cm^−1^, associated with N–H deformations and C–N stretches, confirm the participation of amine groups in the formation of new bonds. The broadening and shifting of the band in the 1200–1100 cm^−1^ region may indicate the formation of single C–N and C–S bonds. At the same time, the C=O peak at 1720 cm^−1^ partially remains, which may be associated with residual carboxyl groups on the surface of GO [40]. However, it should be noted that the Boehm titration method failed to detect carboxyl groups in rGO-TU (Table 3). The ratios of intensities in the frequency ranges of 1000–1200 cm^−1^ and 1500–1700 cm^−1^ changed, which may indicate a change in the ratio of the number of carbonyl groups and hydroxyl groups in the GO and rGO-TU samples. This assumption is supported by the results of the Boehm titration. Thus, the combination of spectral data confirms the formation of covalent bonds between GO and thiourea, primarily due to the reaction of amine groups with aldehyde and ketone groups, forming hydroxyalkylthiourea, which, upon dehydration, transforms into *N*-thiocarbamoylimines, as shown in Scheme 1.

**Figure 4:**
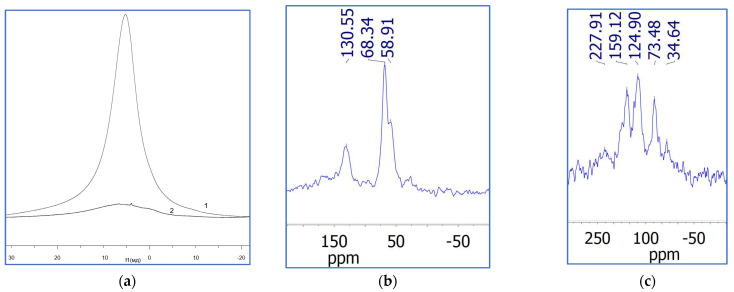
^1^H NMR spectra of GO (1, (**a**)) and aerogel rGO−TU (2, (**a**)), and ^13^C for GO (**b**) and aerogel rGO-TU (**c**).

Solid-state ^1^H and ^13^C NMR spectra recorded for GO and synthesized functionalized materials confirm that GO–imines (Figure 4) have fewer oxygen functionalized groups than GO. In the ^1^H NMR spectrum, the peak at ~5 ppm corresponding to oxygen-containing groups exhibits much lower intensity corresponding to the rGO-TU (2 in Figure 4a) in comparison to the analogous signal in the ^1^H NMR spectrum of GO (1 in Figure 4a). The same conclusion can be drawn from the ^13^C NMR spectra, since peaks in the region of 50–70 ppm, which are assigned to epoxy and hydroxy groups [41,42], exhibit much lower intensity in the corresponding ^13^C NMR spectrum of rGO-TU (Figure 4b) in comparison to the ^13^C NMR spectrum of GO (Figure 4c). The relative intensities of peaks around 20 and 30 ppm, attributed to aliphatic carbon atoms in the CH_2_ and CH_3_ groups in the rGO-TU spectra, indicate the presence of aliphatic hydrocarbon groups in the structure of reduced graphene oxide. Such groups may arise as a result of reduction and be located at defect sites in graphene sheets. Thus, these data indicate the removal of oxygen-containing groups originally present in GO.

Another interesting point that can be observed in the spectra shown in Figure 4 is the upfield chemical shift of the peak associated with sp^2^ carbons for the GO sample, reaching 125 ppm in the case of the GO-TU sample, compared to GO, where the corresponding chemical shift is 130 ppm. This result is similar to that previously obtained for GO functionalized with sulfur-containing groups, where this chemical shift was attributed to an increase in disorder in the basal planes due to the incorporation of functional groups [41,42].

It is interesting to note the appearance of ^13^C NMR peaks in the range of about 140–230 ppm in rGO-TU samples. According to [42], the peak at 158 ppm may be attributed to sp^2^ carbon atoms bound to nitrogen. However, a recent report [43] claims that the signal in this region arises from carbon atoms of carboxylic acid groups. This statement is consistent with the data of study [44], which also assumed that the peaks in this region correspond to carbon atoms in ketone groups. In addition, as noted above, the IR spectra of rGO-TU samples (Figure 3b) demonstrate a signal at 1720 cm^−1^, which also corresponds to the carbon–oxygen bond in the carbonyl atom of the COOH groups. Thus, this gives additional grounds to assume that reaction Scheme 1, which shows the interaction of the amino group of thiourea with carbonyl groups on graphene sheets to form an imine bond, and not with carboxyl groups to form an amide bond. In addition, this assumption is confirmed by the presence of a signal at 226 ppm in the ^13^C NMR spectrum of the rGO-TU sample. According to [45], the carbon atom in the C=S bond, in the immediate vicinity of which there is an imine bond, provides a signal at 220 ppm. Thus, it can be concluded that the reduction of graphene oxide occurs in parallel with the functionalization of its oxygen-containing groups, providing additional cross-linking of the graphene sheets of the reduced graphene.

**Figure 5:**
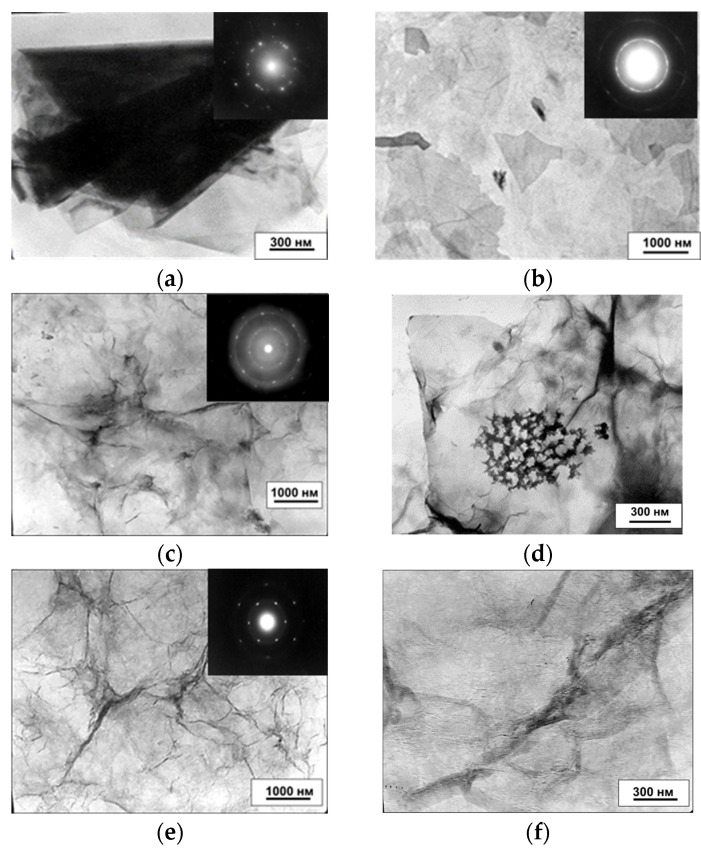
Transmission electron microscopy (TEM) images and TEM electron diffraction pattern of GO (**b**); hydrogels obtained by reduction of GO with thiourea (**c**) and ascorbic acid (**e**) ((**a**)—graphite; (**b**)—GO; (**c**,**d**)—rGO-TU, (**e**,**f**)—rGO-AA).

The results of the surface examination of GO samples and the newly obtained hydrogels rGO-TU and rGO-AA using the TEM method are shown in Figure 5.

It can be seen that the clear diffraction reflections characteristic of graphite (Figure 5a) and rGO-AA (Figure 5e) became blurred in the GO (Figure 5b) and rGO-TU (Figure 5c) samples. This indicates variations in layers of the graphene in these samples in different regions within the electron beam aperture. In the TEM images, the graphene sheets of rGO-TU have folds. This morphology of rGO can be explained by the bonding between interlayer graphene sheets via different functional groups, as well as the presence of stresses and defects that arise during the formation of the hydrogel.

**Figure 6:**
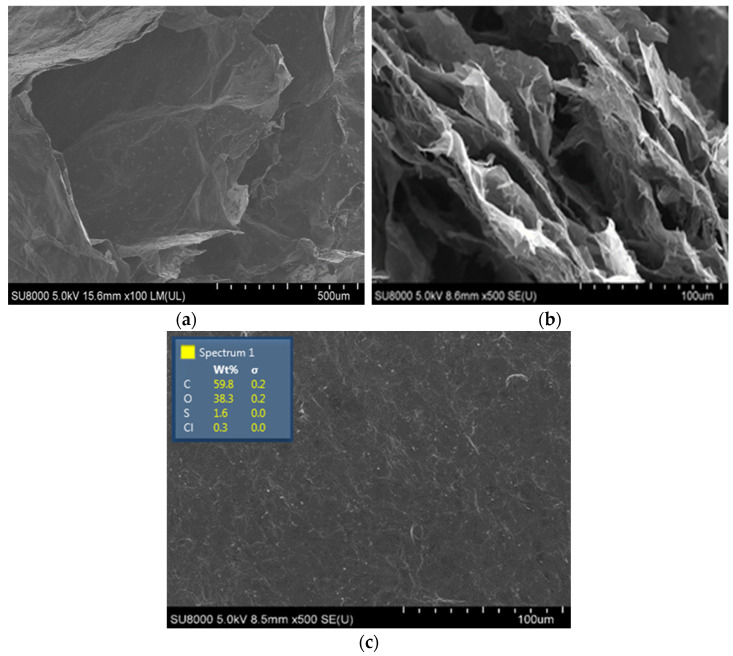
SEM images of aerogels rGO-TU (**a**) and rGO-AA (**b**), and GO (**c**).

Sublimation drying of rGO hydrogels enabled a preparation of aerogels, in which the 3D arrangement of graphene sheets observed in hydrogel samples became fixed. Indeed, according to the SEM results presented in Figure 6, the rGO-TU aerogel samples retained their layered structure. According to the results of the SEM presented in Figure 6, the samples of exhaust gas aerogels obtained during the reduction of TU (Figure 6a) and AA (Figure 5b) retained their layered structure as much as possible.

The distance between the graphite layers for rGO-TU ranged from 5 to 500 µm, and those for rGO-AA from 2 to 100 µm. When analyzing the SEM images of rGO-TU, structural formations were found on the entire surfaces of the graphite sheets. Similar formations are observed on the surfaces of graphene sheets and in TEM images (Figure 5d). According to the TEM data, the area of inclusions is approximately 500 nm. Deposits are observed mainly in the folds of graphene sheets. No such formations were found on the surface of rGO-AA. It was suggested that the detected microcrystals may be thiourea.

**Figure 7:**
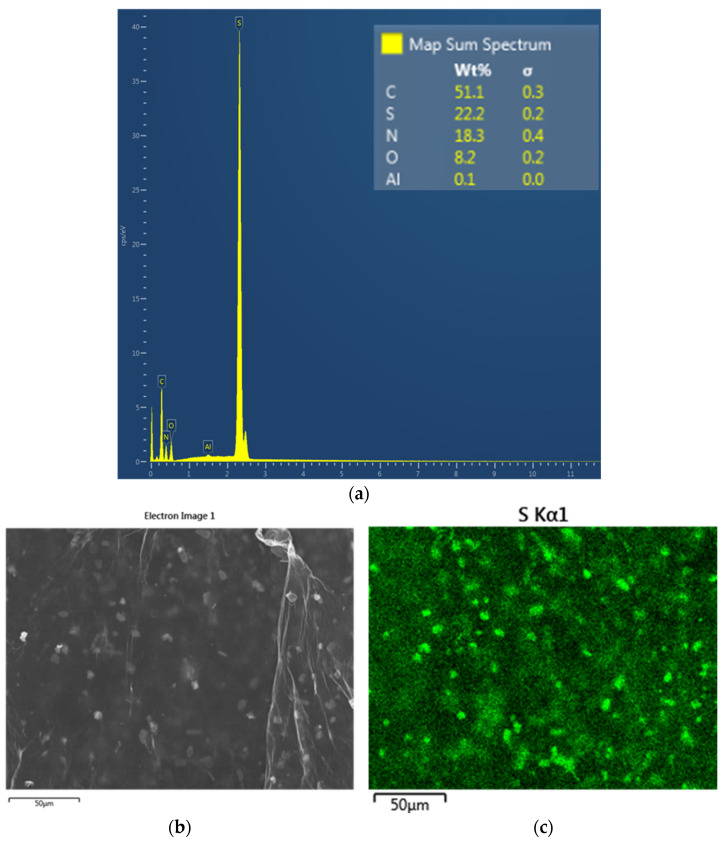
EDS spectrum and elemental map (**a**) according to SEM data from the surface of rGO-TU (**b**,**c**).

This assumption is confirmed by the elemental SEM map of the rGO-TU surface (Figure 7).

The percentage ratio of the atomic masses of sulfur and nitrogen corresponds to their ratio in the thiourea molecule W_t%_S/W_t%_N = 1.2, and it is the presence of thiourea crystals that can produce pronounced blurred diffraction reflections in the electron diffraction images of rGO-TU hydrogels (Figure 5d). In addition, the absorption band in the IR spectrum of rGO-TU at ~730 cm^−1^, characteristic of C=S or N–C=S fragments, additionally indicates a residual thiogroup in the composition of rGO-TU [46].

According to the SEM-EDS data, a significant amount of oxygen is present in the rGO samples, which indicates the absence of complete graphene oxide reduction. Sulfur in the GO samples and their reduced forms indicates the presence of residual sulfur-containing groups on the surface of graphene sheets after graphite oxidation with sulfuric acid. It is interesting to note that during the reduction of GO with thiourea, the sulfur content increased significantly, while for the rGO-AA samples, the sulfur content decreased. It should also be added here that the amount of oxygen in the rGO-AA samples decreased slightly compared to GO. At first glance, this may be due to insufficient GO recovery. However, an analysis of the results of IR spectroscopy and Boehm titration suggests that a residual amount of ascorbic acid is present in the rGO-AA aerogel sample. Thus, the intensity of the peak at 1735 cm^−1^ increased on the FTIR spectrum, corresponding to carboxyl groups of ascorbic acid (Figure 3c). The Boehm titration results (Table 4) also confirm the high content of carboxyl and hydroxyl groups in rGO-AA.

### 3.2. Adsorption Properties

**Figure 8:**
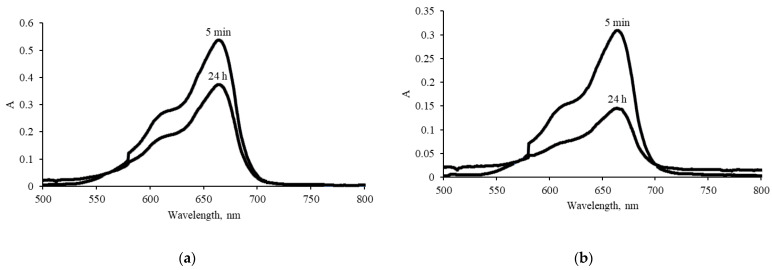
UV–vis absorption spectra after various contact times of MB with aerogel rGO-TU (**a**) and rGO-AA (**b**).

Two types of measurements were carried out to determine the adsorption capacity of the reduced GO with respect to MB. The first determination of the residual concentration of MB was carried out after 5 min of shaking the aerogel/MB suspension, and the second after settling the reaction mixture for 24 h (Figure 8).

The sorption capacities of rGO-TU aerogel are 53.5 mg/g and 60.2 mg/g, respectively. These values are lower than those given in [47], in which the reduction of GO was carried out using the hydrothermal method and for simple rGO-AA (Table 5). The obtained low values of sorption capacity are indirect evidence of a decreased number of sorption centers on the surface of rGO due to their binding with thiourea that did not react.

## 4. Conclusions

Thus, in this study, graphene oxide samples reduced with thiourea and ascorbic acid were obtained. The reduction efficiency was confirmed by X-ray diffraction, FTIR, and Raman spectroscopy. Three-dimensional carbon materials (aerogels)—rGO-TU and rGO-AA—were obtained from hydrogels of reduced GO by sublimation. The morphology of the hydrogels and aerogels was characterized by SEM and TEM. According to SSNMR, FTIR, Boehm titration, and elemental analysis, the reduction of graphene oxide with thiourea results in a reduction of oxygen-containing groups. However, for samples of graphene oxide reduced with ascorbic acid, the FTIR, Boehm titration, and elemental analysis data cannot be considered reliable due to the presence of reducing agent residues. The methylene blue sorption capacity of the aerogels was measured with pH and temperature control. The methylene blue adsorption capacity of rGO-TU aerogel after 1440 min was 60.2 mg/g, while for rGO-AA it was 71.41 mg/g. However, the use of thiourea as a chemical reducing agent for GO has several unique features. On the one hand, the reduction of graphene oxide occurs with the simultaneous functionalization of its oxygen-containing groups. On the other hand, thiourea interacts with the GO surface, likely through electrostatic interactions and hydrogen bonds. This apparently reduces the sorption properties of rGO-TU compared to rGO-AA. Interestingly, thiourea molecules are localized and concentrated on the GO surface in the folds of graphene sheets, allowing their identification by SEM and TEM. These results introduce some limitations in the use of thiourea as a reducing agent in graphene material production technologies.

## Figures and Tables

**Table 1:** X-ray diffraction data for graphite, GO, rGO-TU, and rGO-AA.

Sample	(002) Diffraction Peak				
	2θ, °	FWHM, °	H, nm	d, nm	n
Graphite	26.60	0.32	26.66	0.34	78–79
GO	11.41	0.61	13.50	0.80	16–17
rGO-TU	23.24	5.58	1.52	0.38	4
rGO-AA	19.68	5.52	1.53	0.45	3–4

**Table 2:** Raman spectroscopy data for graphite, GO, and rGO-TU.

Sample	Frequency, cm^−1^		Intensity (I)		FWHM		I_D_/I_G_	L_a_, nm
	Band D	Band G	Band D	Band G	Band D	Band G		
Graphite	1333	1586	106	731	39.97	14.99	0.15	265.44
GO	1355	1589	101	101	80.75	76.39	1.00	38.48
rGO-TU	1322	1588	629	201	82.89	64.68	3.13	12.99

**Table 3:** Surface characteristics of the carbon materials obtained using the Boehm method.

Sample	Total Acidic Groups, mmol/g	Carboxyl	Lactone	Phenol
GO	1.90	0.10	1.42	0.38
rGO-TU	0.41	-	0.39	0.02
rGO-AA	2.15	0.42	1.15	0.58

**Table 4:** The elemental composition of GO and its reduced forms.

Element	Percentage of the Element, %		
	GO	rGO-TU Aerogel	rGO-AA Aerogel
S Ka	1.6 ± 0.0	6.49 ± 1.75	0.12 ± 0.03
O Ka	38.3 ± 0.2	24.73 ± 3.25	33.26 ± 1.91
N Ka	-	21.94 ± 5.60	-
C Ka	59.8 ± 0.2	46.84 ± 8.33	66.62 ± 1.88
Cl Ka	0.3 ± 0.0	-	-

**Table 5:** Adsorption capacity of rGO aerogels with respect to MB.

Parameter	rGO-TU Aerogel		rGO-AA Aerogel	
	in 5 min	in 1440 min	in 5 min	in 1440 min
C_in_, mg/L	4.26	2.92	4.08	1.09
X, mg/g	53.47	60.20	56.01	71.41

## Data Availability

The original contributions presented in this study are included in the article. Further inquiries can be directed to the corresponding author.
